# Population structure and invasion history of *Aedes aegypti* (Diptera: Culicidae) in Southeast Asia and Australasia

**DOI:** 10.1111/eva.13541

**Published:** 2023-03-25

**Authors:** Andrew J. Maynard, Luke Ambrose, Michael J. Bangs, Rohani Ahmad, Charles Butafa, Nigel W. Beebe

**Affiliations:** ^1^ School of Biological Sciences University of Queensland Brisbane Queensland Australia; ^2^ Public Health and Malaria Control Program International SOS and PT Freeport Indonesia Papua Indonesia; ^3^ Medical Entomology Unit Institute of Medical Research Kuala Lumpur Malaysia; ^4^ National Vector Borne Diseases Control Program Ministry of Health and Medical Services Honiara Solomon Islands; ^5^ CSIRO, Dutton Park Brisbane Queensland Australia

**Keywords:** *Aedes aegypti*, Indo‐Pacific, microsatellites, mtDNA, population genetics – empirical

## Abstract

The dengue mosquito, *Aedes aegypti* (Linnaeus, 1762), is a highly invasive and medically significant vector of dengue, yellow fever, chikungunya and Zika viruses, whose global spread can be attributed to increased globalization in the 15th through 20th century. Records of the invasion history of *Ae. aegypti* across Southeast Asia are sparse and there is little knowledge regarding the invasion routes that the species exploited to gain a foothold in the Indo‐Pacific. Likewise, a broad and geographically thorough investigation of *Ae. aegypti* population genetics in the Indo‐Pacific is lacking, despite this region being highly impacted by diseases transmitted by this species. We assess 11 nuclear microsatellites and mitochondrial *COI* sequences, coupled with widespread sampling through the Indo‐Pacific region to characterise population structure at a broad geographic scale. We also perform a comprehensive literature search to collate documentation of the first known records of *Ae. aegypti* at various locations in the Indo‐Pacific. We revealed additional spatial population genetic structure of *Ae. aegypti* in Southeast Asia, the Indo‐Pacific and Australasia compared with previous studies and find differentiation between multiple Queensland and Torres Strait Islands populations. We also detected additional genetic breaks within Australia, Indonesia and Malaysia. Characterising the structure of previously unexplored populations through this region enhances the understanding of the population structure of *Ae. aegypti* in Australasia and Southeast Asia and may assist predictions of future mosquito movement, informing control strategies as well as assessing the risk of new invasion pathways.

## 1 INTRODUCTION

The dengue mosquito, *Aedes aegypti* (Linnaeus, 1762), is a polymorphic species native to Africa. Belonging to a species group known as the Aegypti Group, whose diversity is centred in the islands of the Indian Ocean, it is thought to have initially reached mainland Africa less than 100,000 years ago (Soghigian et al., 2020). At this stage of its history, *Ae. aegypti* (subspecies *formosus*) blood‐fed on non‐human hosts and inhabited tropical forests (Brown et al., 2014; Mattingly, 1957; Soghigian et al., 2020). However, a combination of recent changes in human water storage practices and climate has resulted in the evolution of a highly anthropophilic subspecies, *Aedes aegypti aegypti*, which uses artificial water‐holding containers as larval habitat (Brown et al., 2014; Rose et al., 2020). The evolution of anthropophily in *Ae. aegypti* has dramatically increased its disease‐transmitting potential (Ritchie, 2014), and its use of artificial containers as larval habitat and desiccation‐resistant eggs has contributed to its successful invasion of most of the world (Brown et al., 2014). With a widespread distribution in urbanised areas of the tropics and subtropics, it is a major pest and acts as the principal vector of dengue, chikungunya and Zika viruses (Rose et al., 2020). Although the broadscale global invasion history and population genetics of *Ae. aegypti* has been studied extensively, finer scale invasion pathways and population structure in some regions are less well known. In this study, we focus on improving knowledge of population structure and invasion history of *Ae. aegypti* in Southeast Asia, particularly the Indo‐Pacific.

It is likely that *Ae. aegypti* first established in Asia in the late 19th century, coinciding with the first reports of dengue fever from an urban setting where *Ae. aegypti* was the suspected vector (Smith, 1956). In Southeast Asia, the first evidence for the establishment of the species is at major ports around the Malaysian Peninsula in Singapore in the early 1900s, Fontaine (1899) quoted by Thoebald (1901), Port Klang (Malaysia) (Daniels, 1908) and Indonesia (Java, Sumatra and Sulawesi, 1901–1916) Marlatt quoted by Howard et al. (1917), (Boyce, 1911; Schüffner & Swellengrebel, 1914; Stanton, 1920), before spreading along the coast and then inland. Other regions of Asia showed mostly coastal distributions or only establishment at major ports, suggesting later introductions in regions including Thailand, Vietnam, India, Myanmar and China (Farner et al., 1946; Kumm, 1931; Theobald, 1911). Ports in the Bay of Bengal could have acted as an important introduction pathway into Asia given its strong history of trade; although the first occurrence records were from 1899 in India; Goodrich (1899) and James (1900) quoted by Theobald (1901) and 1901 in Upper Myanmar (Watson quoted by Theobald, 1901). Recent molecular studies examining the global population genetics of the species have suggested that the ‘Asian’ (including some populations from the Pacific and Australia) invasion of *Ae. aegypti* was most likely seeded from the Americas rather than from an African source (Brown et al., 2014; Gloria‐Soria et al., 2016; Powell & Tabachnick, 2013). Other such studies present conflicting results and pathways (West Africa to Asia to the Americas; see Bennett et al., 2016), albeit with low confidence in results. However, these studies were performed at a global scale and combined broad geographic regions to simplify invasion scenarios.

In Australia, reports of dengue suggest the species established at a similar time as in the Asian region, possibly prior. The first indigenous outbreaks of dengue in Australia occurred in Townsville, Queensland (QLD) in 1879 and later in Rockhampton, QLD in 1885 (Lumley & Taylor, 1943a), with several epidemics later described during the 1890s and early 20th century (Mackenzie et al., 1996). Records of urban endemicity of dengue commenced in India and Indonesia from the late‐1800s to early‐1900s for mainland Southeast Asia (Smith, 1956), although little interest was paid to dengue during this time. Unlike the recent arrival of *Aedes albopictus* into northern Australia which appears to have originated from Indonesia (Beebe et al., 2013; Maynard et al., 2017), genetic evidence (SNP and nuclear gene sequences) suggests the older invasion by *Ae. aegypti* into Australia was likely from an Asian, American or Western Pacific source (Gloria‐Soria et al., 2016; Powell & Tabachnick, 2013). The possibility of the Mediterranean acting as an invasion source following the opening of the Suez Canal has also been suggested (Powell et al., 2018).

The first specimen of *Ae. aegypti* in Australia was recorded from the remote inland Queensland town of Cunnamulla in 1881 (Lumley & Taylor, 1943b; Taylor, 1915a), shortly followed by a record from Brisbane in 1887 (Skuse, 1889). Once in Australia, *Ae. aegypti* spread rapidly via rail (Hamlyn‐Harris, 1927), both inland and along the coast. Since then, its distribution has decreased substantially due to reduction in rainwater tanks in the second half of the 20th century (Beebe et al., 2009; Trewin et al., 2017). Today, it is only found in Queensland, where it is responsible for occasional outbreaks of dengue fever in northern regions. Past studies have shown that the Torres Strait Islands' population of Waiben are mitochondrially distinct from other northern Queensland populations (Beebe et al., 2005).

Records of *Ae. aegypti* in New Guinea start in 1907 (Theobald, 1907), and more specifically noted in Friedrich Wilhelmshafen (now Madang, Papua New Guinea) and Dorey (now Manokwari, West Papua, Indonesia) later in 1910 (Walker & Biro quoted by Theobald, 1911). *Aedes aegypti* was also found on steamers, De Rook quoted by Bonne‐Wepster and Brug (1932) travelling to Tanah Merah (southern Netherlands New Guinea) and at various locations in the New Guinea region between 1910 and 1930s (Hill, 1925; Howard et al., 1917; Stanton, 1920; Taylor, 1914, 1915b; Theobald, 1911). Farner et al. (1946) noted the species' distribution was somewhat discontinuous in New Guinea and limited to areas connected through sea and river traffic (Farner et al., 1946); a pattern which was still apparent in 1987 (Lee et al., 1980), reflecting the species' strong ties to human movements.

**FIGURE 1:**
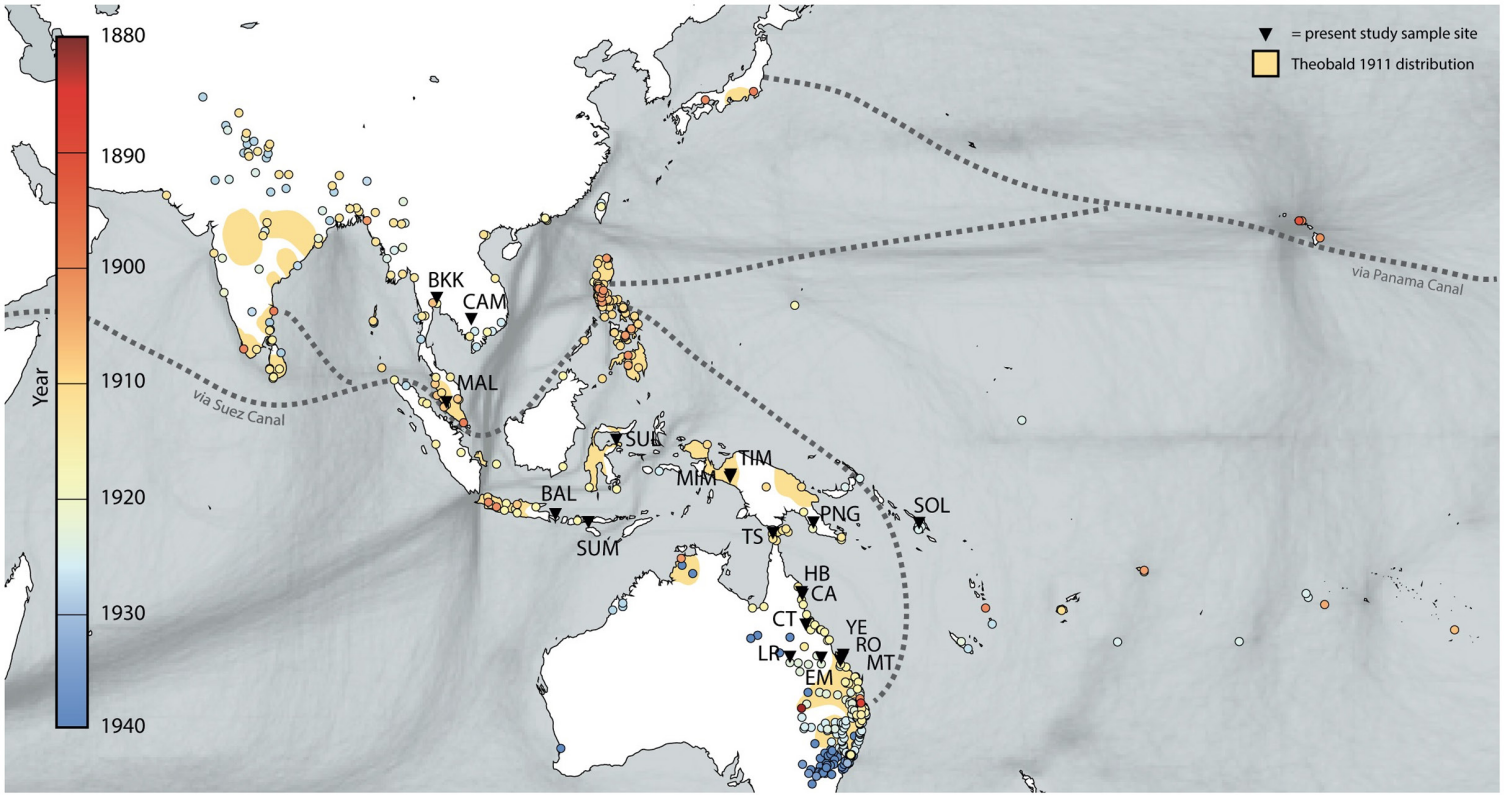
Sample sites of the present study and invasion history of *Aedes aegypti* with regards to historical presence records. This figure does not necessarily reflect the actual date of invasion, but provides an overview based on some of the first records of *Ae. aegypti* in the study region (1880–1940). Circles indicate occurrence records and are colour‐coded based on timing (corresponding to the timeline [left]). Black triangles represent sample sites in the present study (refer to Table 1 for population names; Tucson, Arizona population excluded from the figure). The yellow‐shaded area represents an early distribution map of *Ae. aegypti* by Theobald (1911) which has been modified slightly to fit the current map and to correct Australian records from Southern Australia (which were unreliable: see Lee et al., 1980). Dashed dark grey lines show major shipping routes as a result of the opening of the Suez and Panama canals, while lighter grey lines show the density of shipping movements between 1784 and 1863 (US Maury Collection; modified from Ben Schmidt). For plotted records see Table S1.

To the east, in the Nggella Islands of the Solomon Archipelago, *Ae. aegypti* was common in the houses of Tulagi (the then capital city) Garment quoted by Edwards (1925), Ferguson (1923) and the nearby Purvis Bay in 1925; White quoted by Buxton (1927). This suggests that the species began to establish in the Solomon Islands around the 1920–1930s. Troop movements into the Pacific Islands during World War II are likely to have greatly expanded the distribution of *Ae. aegypti* and contributed to the dispersal between geographically distant and genetically distinct populations (Calvez et al., 2016; Failloux et al., 2002). For an overview of occurrence records within Southeast Asia and Australasia refer to Figure 1 and Table S1. Overall, the records presented in the figure show that the first recorded appearances of *Ae. aegypti* occurred rapidly and at a similar timeframe at the turn of the 20th century (Figure 1). These correspond broadly with urban dengue and chikungunya records in the region (Carey, 1971; Mackenzie et al., 1996; Smith, 1956). By the 1940s, *Ae. aegypti* was ubiquitous in the tropics of the region (see Farner et al. (1946) distribution map), but remained absent from certain areas (Kraemer, Sinka, Duda, Mylne, Shearer, Barker, et al., 2015; Kraemer, Sinka, Duda, Mylne, Shearer, Brady, et al., 2015).

In this study, we aim to first document the invasion history of *Ae. aegypti* in the Indo‐Pacific region from historical literature and secondly further investigate the population structure of *Ae. aegypti* in the Indo‐Pacific region by including previously unstudied populations and geographic areas. We hypothesise that the population structure of *Ae. aegypti* is characterised by both isolation by distance and historical and contemporary human transportation routes. We expect that the species' invasion into Southeast Asia and Australasia involved a history of multiple, independent introductions and that this will be reflected in our population genetic data.

## 2 MATERIALS AND METHODS

### 2.1 Sampling and species identification

Our collection sites consisted of 20 populations distributed throughout the Southeast Asia and Australasia region (Figure 1, black triangles). This included populations from Arizona (USA), Australia, New Guinea (Papua New Guinea and Papua‐Indonesia), the Solomon Islands, Indonesia (Bali, Sulawesi and Sumba) and mainland SE Asia (Malaysia, Thailand and Cambodia; Table 1; Table S2). Adult samples were collected between 2008 and 2016 using aspiration/sweep netting while larvae were collected from suitable breeding habitats using dipping. Samples were either stored in 70%–100% EtOH or desiccated (adults) over silica beads. DNA was extracted from samples of *Ae. aegypti* using a salt extraction protocol (Beebe et al., 2005) and diluted at 1:10 in 1 × TE buffer (Tris, EDTA). Species identification was verified through morphology or in difficult cases using PCR‐restriction digest species diagnostic (Beebe et al., 2007).

**TABLE 1:** Sample information for *Aedes aegypti* from Southeast Asia and Australia used in our study.

Region	Population	Code	*n*	Year
Australia (AUS)	Waiben (Thursday Is.), Torres Strait Is.	TS	18	2015
	Holloways Beach	HB	17	2008
	Cairns	CA	26	2008
	Charters Towers	CT	22	2010
	Longreach	LR	26	2011
	Rockhampton	RO	25	2010
	Emerald	EM	27	2010
	Mt Morgan	MT	21	2010
	Yeppoon	YE	12	2010
Pacific (PAC)	Honiara, Guadalcanal, Solomon Islands	SOL	18	2015
	Port Moresby, Papua New Guinea	PNG	8	2014
Indonesia (INA)	Timika, Papua	TIM	16	2013 (6) 2015 (10)
	Amamapare, Mimika, Papua	MIM	10	2015
	Luwuk, Sulawesi	SUL	14	2014
	Waitabula, Sumba	SUM	8	2014
	Kuta, Bali	BAL	27	2015
Malaysia (MAL)	Kuala Lumpur	MAL	35	2015
Mainland Southeast Asia (SEA)	Bangkok, Thailand	BKK	11	2016
	Tro Pang Sap Village, Cambodia	CAM	13	2015
United States of America (USA)	Tucson, Arizona	AZ	12	2015

*Note*: Regional and population definitions are shown, as are population abbreviations used in some figures and text. Sample size (*n*) is also indicated per population. Regional abbreviations that are used in approximate Bayesian computation are shown in brackets in the ‘Region’ column. Further details are in Table S2.

### 2.2 Microsatellite amplification, allele scoring and analysis

Samples were screened for 11 microsatellite markers (Table 2). These markers have been employed in previous population genetic studies on *Ae. aegypti* (Calvez et al., 2016). We attempted to use an additional microsatellite marker (AG2) but found that this consistently failed to amplify in many samples and was thus excluded early in the study. Microsatellites were amplified and tagged with fluorescent dye using M13 tails in 15.4 μL reactions consisting of 10.8 μL H_2_O, 3 μL 5 X Mytaq buffer (Bioline, with pre‐optimised concentrations of dNTPs and MgCl), 0.1 μL 10 μM M13 tagged forward primer, 0.2 μL 10 μM reverse primer, 0.2 μL M13 tagged fluorescent dye (VIC, NED, PET or FAM), 0.01 μL (1 U) MyTaq polymerase and 1 μL of 1:10 DNA template. Subsequent PCR involved denaturation at 96°C for 3 min, followed by 13 cycles of denaturation at 95°C for 30 s, annealing at 56°C for 40 s (with a gradient decrease of 0.5°C/cycle) and extension at 72°C for 30 s. This was followed by a further 25 cycles of 95°C for 30 s, 50°C for 40 s and 72°C for 30 s. Then a final elongation step of 5 min at 72°C before cooling to 4°C. Amplification was confirmed by running 1 μL of the PCR product on a 2% agarose gel stained with MidoriGreen (Bulldog Bio; 1 μL per 100 mL of 2% agarose in 1 × TBE buffer). Successfully amplified samples were sent to Macrogen Inc. (Republic of Korea) for fragment analysis on an ABI 3730XL DNA analyzer (Applied Biosystems, Waltham, Massachusetts, USA).

**TABLE 2:** Microsatellite characteristics for 11 loci screened on *Aedes aegypti*.

Locus	*N*_a_	*N*_e_	*H*_o_	uHe	HW	Null alleles	g *F*_ST_	g *F*_ST_ (no null)
A9	6	3.43	0.29	0.71	4	0.24	0.30	0.25
B2	9	2.05	0.30	0.51	3	0.14	0.24	0.24
AC5	14	3.29	0.55	0.70	3	0.08	0.10	0.09
AG5	9	3.45	0.65	0.71	2	0.04	0.09	0.09
A1	6	2.83	0.55	0.65	1	0.06	0.14	0.13
B3	14	2.87	0.52	0.65	1	0.08	0.13	0.12
AG1	8	4.55	0.60	0.78	1	0.10	0.12	0.11
AC2	6	2.24	0.43	0.56	1	0.07	0.24	0.23
AC4	5	1.73	0.13	0.42	10	0.22	0.16	0.15
AC1	11	3.39	0.56	0.71	3	0.09	0.16	0.16
CT2	9	2.17	0.33	0.54	4	0.13	0.31	0.30

*Note*: Mean number of alleles (*N*_a_), number of effective alleles (*N*_e_), observed heterozygosity (*H*_o_), unbiased expected heterozygosity (uHe), number of populations deviating from Hardy–Weinberg equilibrium (HW), null alleles, global *F*_ST_ (g*F*_ST_) and global *F*_ST_ without null alleles (g*F*_ST_ [no null]) following ENA correction (Chapuis & Estoup, 2006) are displayed for each locus.

Raw microsatellite data were processed using the standardization run wizard (default animal fragment settings) in GeneMarker v.2.4.2 (SoftGenetics LLC; Hulce et al., 2011) and alleles were scored manually. A random selection of genotyped plates was scored by a second person to assess consistency in results. Poor‐quality samples with weak or messy peaks were removed from the final data set due to an excess of missing data; additionally, those with fewer than eight out of 11 scored loci were removed. This left 366 individuals for the final analyses (Table S2).

We replaced missing microsatellite values based on mean population allele frequencies using GenoDive v. 2.0b27 (Meirmans & Van Tienderen, 2004); this adjusted dataset was used to conduct Discriminant analysis of principal components (DAPC) and pairwise genetic distance calculations. Missing values were not replaced for STRUCTURE analyses or the calculation of Hardy–Weinberg equilibrium (HW), linkage disequilibrium and for testing for null alleles. For each locus we calculated allelic richness (*N*_a_), number of effective alleles (*N*_e_), observed (*H*_o_) and unbiased expected values of heterozygosity (uHe) and global *F*_ST_ with and without the exclusion of null alleles using Genepop v.4.2 (Raymond et al., 1995; Rousset, 2008) and FreeNA (Chapuis & Estoup, 2006). We checked for HW (with Bonferroni correction) using GenAlEx v.6.5 (Peakall & Smouse, 2012) whereas LD was checked in Genepop. Pairwise population indices of genetic variation for *F*_ST_, G"_ST_ and Jost's D were calculated between populations in GenAlEx v.6.5 using 9999 permutations and analysis of molecular variance (AMOVA) to assess significance. We tested for isolation by distance with a Mantel test, implemented in the adegenet 2.1.1 package (Jombart, 2008; Jombart & Ahmed, 2011) in R v.3.4.4 (R Core Team, 2018), and using matrices of Edward's genetic distance and Euclidean geographic distances based on 9999 replicates.

Population structure was investigated using the program STRUCTURE v.2.3.4 (Pritchard et al., 2000). Preliminary analyses were conducted to investigate the most probable number of population clusters (*K*) present in the dataset and to explore the effect of models using the admixture and population prior settings. Based on these preliminary analyses, the final analysis was run with the admixture model and using sampling locations as a prior, with *K* ranging from 2 to 22 (20 iterations per value of *K*) with a burn‐in of 100,000 followed by 1,000,000 iterations. The output from the STRUCTURE run was processed in STRUCTUREHARVESTER (Earl & vonHoldt, 2012) to infer the most likely value of *K* using the Evanno ∆*K* (Evanno et al., 2005) and *L*(*K*) methods. In addition, we analysed subsets of the dataset based on these STRUCTURE results (commonly referred to as a hierarchical approach, where distinct clusters are sub analysed in independent STRUCTURE runs to explore any substructure). For these sub‐analyses, we used the same settings and run time, but the value of *K* ranged based on the number of populations being analysed. Final plots were made using pophelper (Francis, 2017). CLUMPAK (Kopelman et al., 2015) was used to assess *K* values for each analysis.

We conducted discriminant analyses of principal components as an alternative approach to examine population structure in our dataset using adegenet. Due to the highly domestic nature of *Ae. aegypti* (Powell & Tabachnick, 2013), group membership was predefined based on sampling location (Table 1; Population) and DAPC was initially conducted on the whole dataset. We performed cross‐validation on the DAPC using a validation set of 10% and a training dataset of 90% with 100 replicates. To avoid overfitting the discriminant functions in DAPC, we considered the optimum number of principal components (*n*.pca = 30) to retain as that being associated with the lowest root mean squared error (RMSE; Jombart & Collins, 2015). Five discriminant functions were retained but only the first three were plotted as these explain the most variance. To assist with the display of results, we additionally plotted population means from the DAPC to highlight patterns and reduce noise in the plots.

To further explore clustering in our dataset given no prior population information (i.e. assuming populations are unknown), we used *K*‐means clustering where the various clustering outcomes were compared using the Bayesian information criterion (BIC). The *K*‐means clustering was performed using the adegenet package (using PCA where all principal components were retained). We used the lowest BIC to infer the optimal *K* value. Inferred group memberships were plotted against actual group (population) membership. We additionally performed this using regional definitions (Table 1; Region) to explore how this affected reassignment of individuals.

### 2.3 Mitochondrial COI amplification and analysis

An approximately 550 bp region of the mitochondrial gene *COI* was amplified using previously used (Beebe et al., 2005) primers (aegCOI‐250F 5′‐TAGTTCCTTTAATATTAGGAGC‐3′ and aegCOI‐800R 5′‐TAATATAGCATAAATTATTCC‐3′) for 117 individuals. Each 16 μL reaction contained 10.4 μL H_2_O, 4 μL 5 × Mytaq buffer (Bioline, with pre‐optimised concentrations of dNTPs and MgCl_2_), 0.2 μL 100 μM forward primer, 0.2 μL 100 μM reverse primer, 0.2 μL MyTaq polymerase and 1 μL DNA template. For PCR, we used an initial denature of 94°C for 3 min, 35 cycles of denaturation at 95°C for 30 s, primer annealing at 45°C for 40 s, and primer extension at 72°C for 30 s. Final elongation was 5 min at 72°C prior to storing at 4°C. Amplification was confirmed using gel electrophoresis (as described previously) and PCR products were purified by adding 2.5 μL per sample of a mixture containing 1.4 μL H_2_O, 1 μL Exonuclease I and 0.1 μL Shrimp Alkaline Phosphatase (rSAP; New England Biolabs, Australia) before incubation at 37°C for 20 min and denaturation at 80°C for 10 min. Samples were sequenced in both the forward and reverse directions by Macrogen Inc. (Republic of Korea) using Sanger sequencing. An automated workflow was used in Geneious v.11.1 (http://www.geneious.com, Kearse et al., 2012) to first trim ends of the sequences (error rate 0.01%), de novo assemble forward and reverse sequences from the same individuals (at which point alignment and chromatogram quality was visually assessed for all sequences) before extracting a consensus sequence for each individual. Sequences that were not processed in the workflow due to either/both forward/reverse reads being low quality were visually inspected; if one read was of acceptable quality (≥65%) then this single read was used to generate a sequence. From the 117 individuals sequenced, 111 sequences were of adequate quality.

Additional *COI* sequences of *Ae*. *aegypti* were obtained from Genbank (810 sequences total, 111 produced in this study, Table S3). Sequences were aligned in Geneious using the MAFFT alignment (Katoh & Standley, 2013). All sequences were trimmed to 335 bp to incorporate the large number of *COI* sequences from Genbank, many of which were smaller than, or did not overlap fully with, the ~550 bp region sequenced in this study. Sequences were checked for stop codons and a TCS haplotype network was constructed in PopArt v.1.7 (http://popart.otago.ac.nz) using 1000 iterations.

## 3 RESULTS

### 3.1 Genetic diversity and differentiation (*F*_ST_, Jost's *D* and G"_ST_)

We observed the lowest heterozygosity (*H*_o_ = 0.428) in the Waiben population despite it having the highest mean number of alleles (15.667) within the Australian region and the highest unbiased expected heterozygosity (0.592). Overall, SE Asian populations exhibited high allelic diversity (*N*, *N*_a_ and *N*_e_) while most mainland Australian populations showed comparatively lower measures. Within Australia, Cairns showed the highest mean observed heterozygosity (although standard error overlaps with Waiben), while all other QLD populations had lower, although similar, values of unbiased heterozygosity.

The loci AC4 and A9 were removed due to the high frequency of null alleles (>0.2) and the number of populations that violated HW (Table 2). A total of 86 alleles were recorded across all nine remaining loci (not including AC4 and A9). We found no significant evidence for linkage disequilibrium between any pairs of loci in each of the 20 populations (after Bonferroni correction [α = 0.05]). Allelic richness (*N*_a_) was highest in Kuala Lumpur, Cambodia and Honiara, whereas Port Moresby exhibited the lowest (Table S5). Port Moresby and Timika had the lowest observed heterozygosity, whereas Tucson, AZ had the highest of all populations in our study (Table S5). Overall, the mantel test showed a significant relationship between geographic distance and genetic distance (*y* = 0.0001*x* + 5.2284; *p* = 0.0002), but the correlation is weak (*R* ^2^ = 0.009), implying the influence of other factors shaping the genetic structure of *Ae. aegypti* in the study region.

**FIGURE 2:**
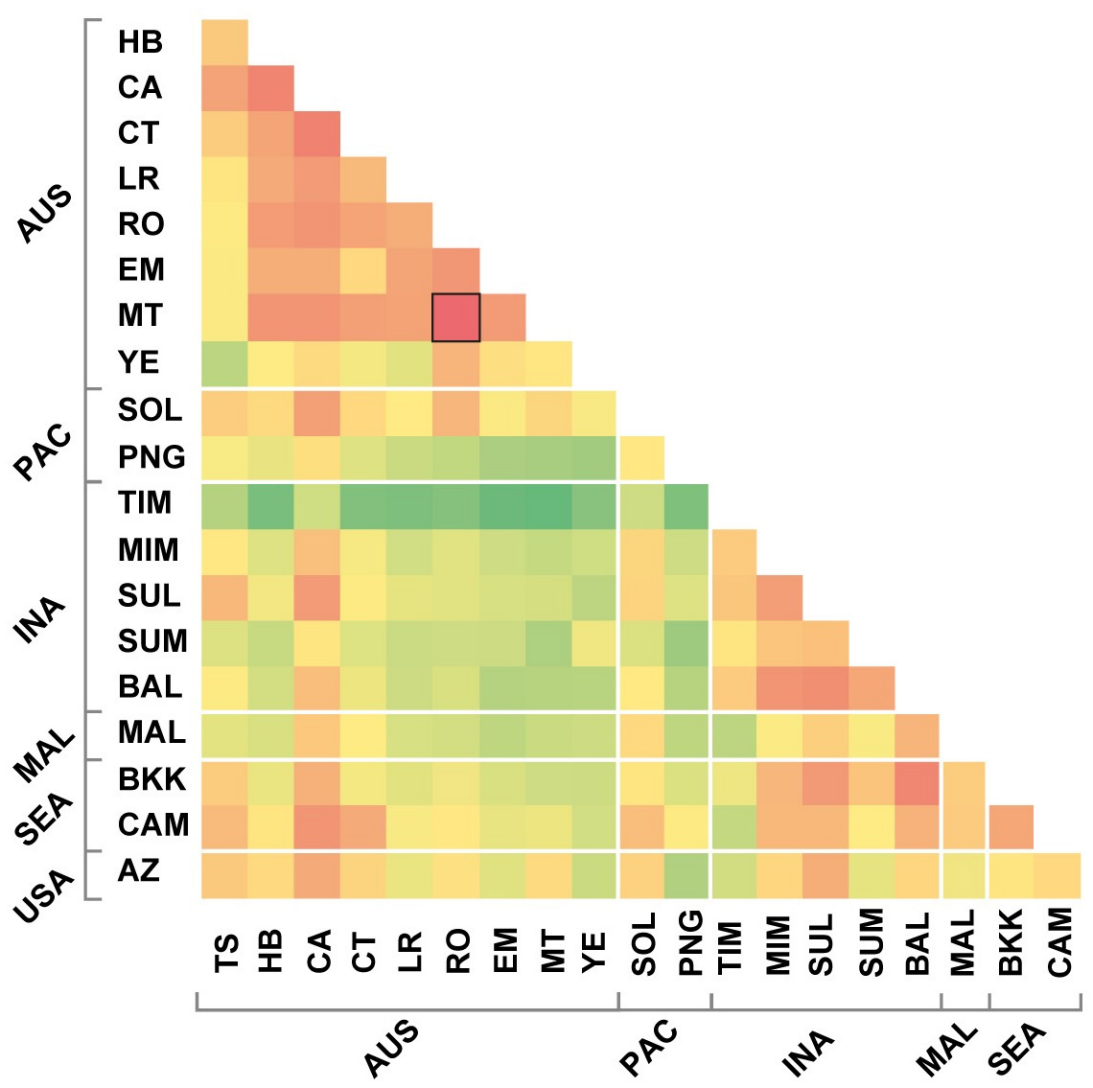
Pairwise genetic distance (*F*_ST_) for all populations of *Aedes aegypti* in the study region. The range of *F*_ST_ is indicated by a colour scale, where redder values indicate a lower *F*_ST_ (min = 0.017) and greener indicates a higher *F*_ST_ (max = 0.249). Insignificant (*p* > 0.05) comparisons are black‐bordered. White lines divide broader regional levels for visualisation purposes. Refer to Table 1 for population abbreviations and Table S5 for *F*_ST_ values.

There was no major difference in the relationships observed using multiple measures of genetic distance (Table S5; *F*_ST_, Jost's *D* and G"_ST_). Overall, significant *F*_ST_ ranged from 0.029 to 0.249 (*p* < 0.005) for the entire dataset. Genetic differentiation was lowest within Indonesia and Australia. Within Queensland, *F*_ST_ ranged from 0.035 to 0.161 (Figure 2). For Waiben of the Torres Strait, Australia, lowest *F*_ST_ scores were observed between nearby Northern Queensland populations (*F*_ST_ = 0.054–0.079; see HB, CA and CT), whereas more southerly populations showed signs of higher genetic differentiation (*F*_ST_: 0.094–0.161; see YE, RO, EM, MT, LR). The New Guinean populations of Port Moresby and Timika displayed high differentiation with most other populations in pairwise comparisons.

### 3.2 Population structure—microsatellites

**FIGURE 3:**
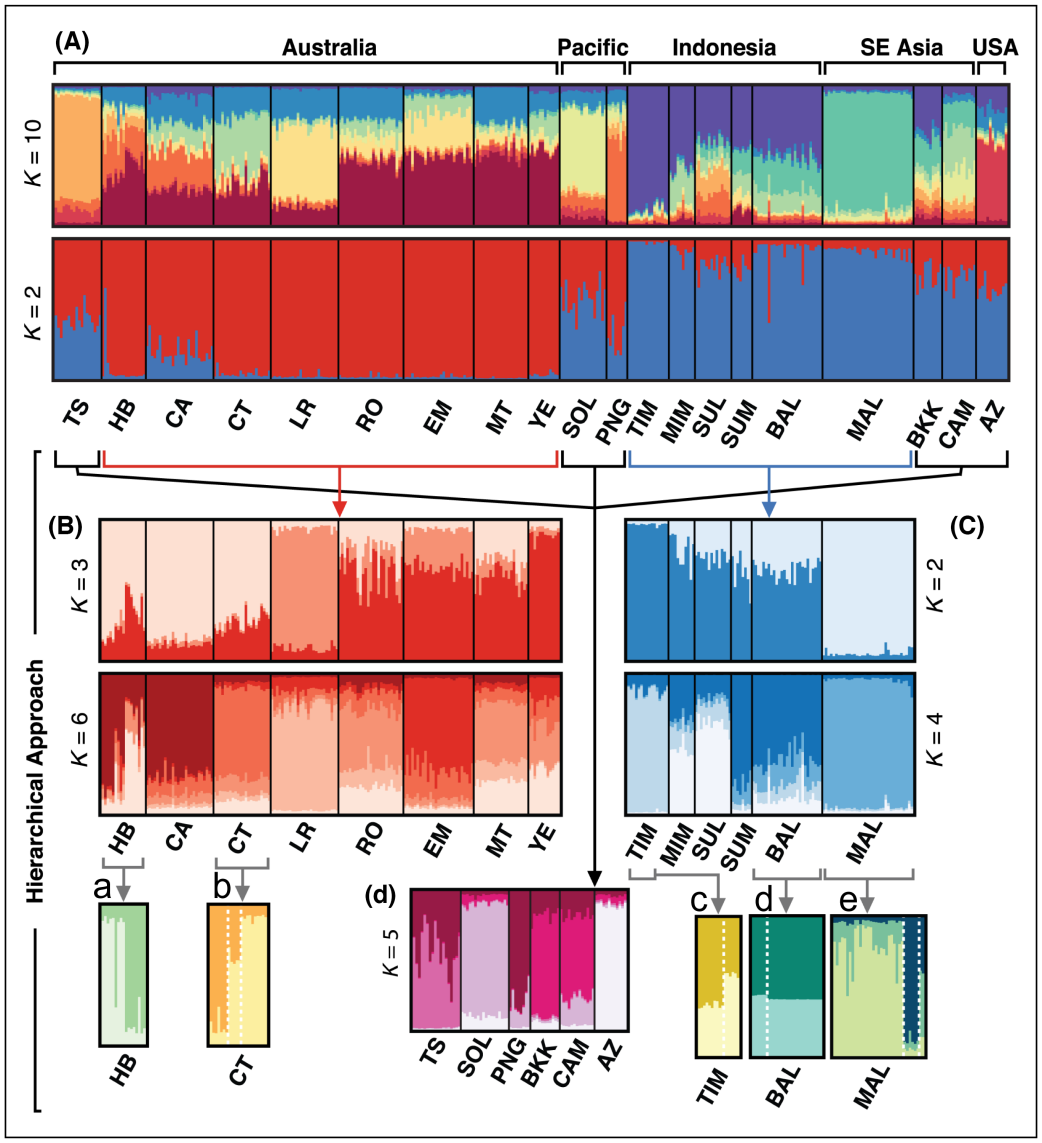
STRUCTURE plots of nine microsatellite loci for 366 samples of *Aedes aegypti* from Southeast Asia and Australasia. Data were analysed as a whole (A) and using a hierarchical approach (B–D) and various values of *K* are displayed. Each vertical bar represents an individual where the bar colour is proportional to genetic cluster membership. Region and population abbreviations are shown in Table 1. White‐dashed lines show sampling site differences within a population (B.b, C.c, C.d, c.e). Arrows show the progressive sub‐analysis of clusters used in the hierarchical approach.

*Aedes aegypti* shows a clear spatial genetic structure in our study region. Using STRUCTURE (Figure 3), populations were more clearly differentiated without the admixture model and using sampling locations as a prior, which assists with clustering when population structure is somewhat weak (Pritchard et al., 2009). However, here we present and discuss results using the admixture model and location prior settings as it provides a more realistic depiction of the population processes occurring within this highly anthropophilic species, which has likely experienced on‐going human‐mediated dispersal and hence admixture between populations.

For the whole dataset, ∆*K* suggested two genetic clusters in the study region (Figure 3). At *K* = 2, the clusters generally correspond to an Australian (red) and Indonesian/Malaysian (blue) clusters with several other populations showing varying degrees of potential admixture between these two clusters (Torres Strait Islands, Solomon Islands, Port Moresby, Bangkok, Cambodia and Arizona). An upper *K* value of 10 (Figure 3) was suggested using median values of Ln(Pr) in CLUMPAK; at this value of *K* populations can be more differentiated and specific clusters dominate different geographic regions, leading to the broad differentiation of mainland Australia, the Torres Strait Islands (Waiben), the Solomon Islands, Papua New Guinea, Indonesia, Malaysia, Southeast Asia and the USA.

To investigate this substructure, a hierarchical approach was used to analyse the red, blue and admixed clusters separately when *K* = 2 on the whole dataset. The Australian cluster (not including Waiben) is made up of approximately 3–6 genetic subgroups (Figure 3A). In general, at *K* = 3 these clusters represent Northern (Cairns, Holloways Beach, Charters Towers), Central (Longreach) and Central‐Eastern (Rockhampton, Mt Morgan and Yeppoon) Queensland divisions. However, there is significant admixture between many of these populations and further differentiation when *K* = 6. Finer population substructure was also uncovered in Holloways Beach (*K* = 2) and Charters Towers (*K* = 2; Figure 3).

Within the blue cluster (Figure 3C), there are approximately 2–4 subgroups. When *K* = 2, Malaysia can be distinguished from the Indonesian populations. Furthermore, when *K* = 4, subgroups appear within Indonesia. These represent Timika, Amamapare (MIM)/Sulawesi and Bali/Sumba. Malaysia was comprised of three genetic clusters when sub‐analysed (Figure 3C), corresponding to differences in sampling site. When admixed populations (<80% overall population membership to either red or blue clusters when *K* = 2) were analysed together, the optimal *K*‐value ranged from 4 to 7. When *K* = 4, genetic clusters correspond to broad geographic groupings: Torres Strait, Papua New Guinea/Solomon Islands, Southeast Asia and the USA.

**FIGURE 4:**
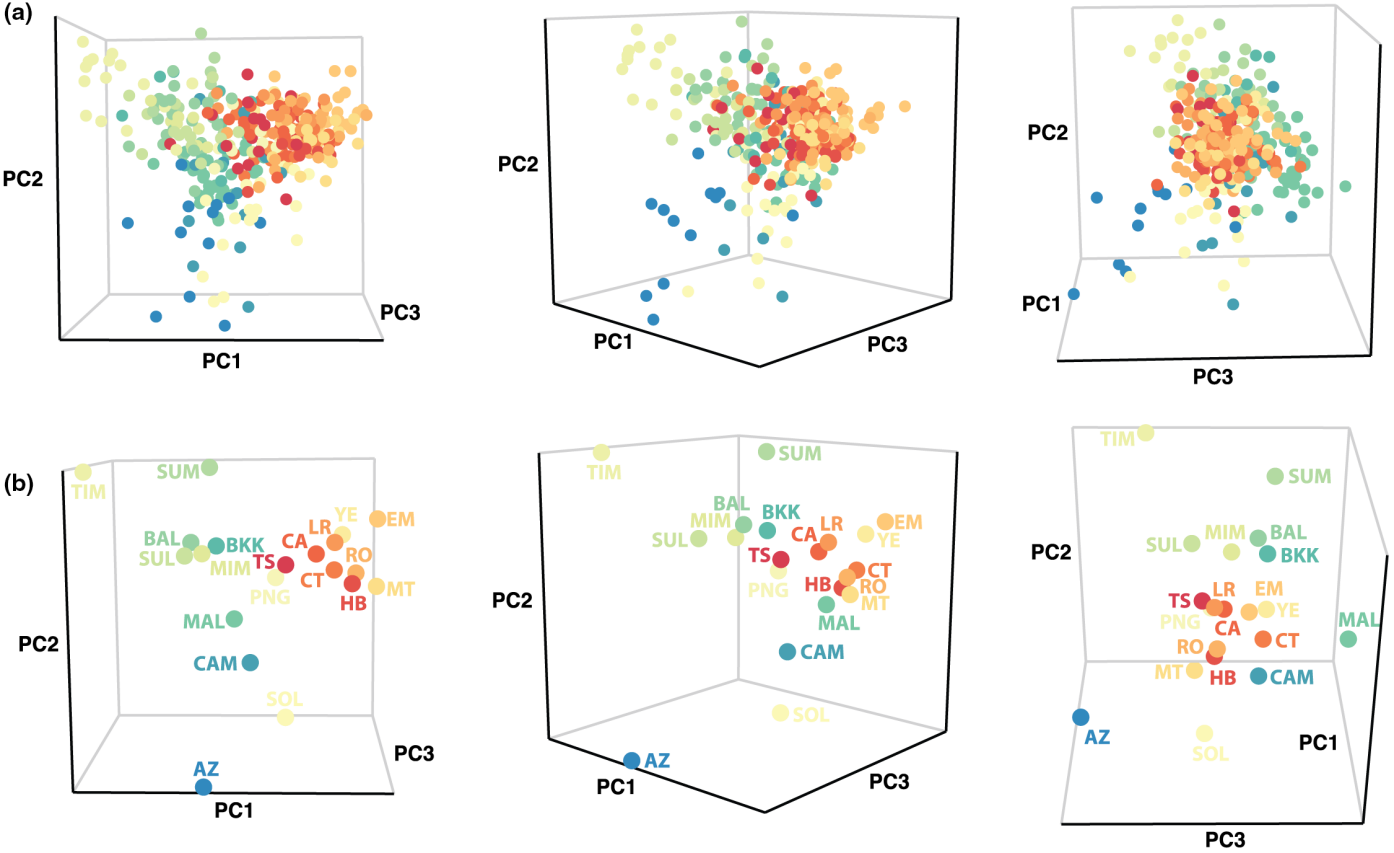
Discriminant analysis of principal components for *Aedes aegypti* (*n* = 366) in our study region using nine microsatellite loci. Principal components 1–3 (PC1‐3) are plotted showing individual variation within population (a) and population means (b). Populations are colour‐coded, and abbreviations are in Table 1.

Discriminant analysis of principal components of the full dataset (Figure 4) is somewhat consistent with the results of the STRUCTURE analysis. In general, there are two main clusters of individuals representing Australia and Indonesia, respectively, with other populations such as Arizona and Timika appearing more differentiated from these. Compared to STRUCTURE results, genetic structure using DAPC appears more conservative (i.e. less substructure revealed). To investigate consistency in results with the hierarchical approach in STRUCTURE, we analysed the same three broad sub‐datasets using DAPC (Figures S1–S3) and a similar genetic structure was observed. When using no population information, approximately 12 genetic clusters were supported by the BIC, where several regions could be genetically distinguished and comprised of several genetic clusters (e.g. inferred clusters 1, 2, 3, 7 and 11 are mostly made up of Australian populations), whereas some clusters are comprised of a mix of multiple regions (e.g. inferred clusters 8 and 12; Figure S4). When the data were analysed using DAPC with broader regional definitions rather than using population information, individuals were mostly assigned to their original cluster with a few exceptions. The proportion of correct reassignment to the original population was 0.41 when populations were used compared to 0.85 when broad regions were used (Figure S5).

### 3.3 Population structure—Mitochondrial *COI*

**FIGURE 5:**
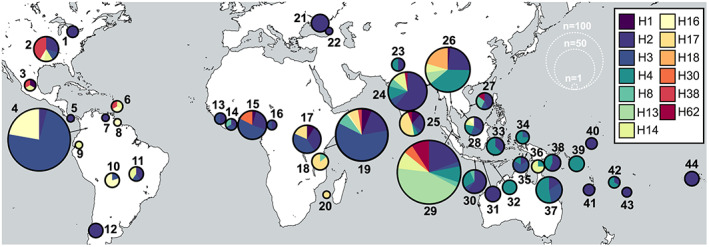
Thirteen of the most prevalent *COI* haplotypes for *Aedes aegypti* on a global scale. Circle size corresponds to the number of sequences (see white dashed scale) from a given locality. The proportion of individuals belonging to a given haplotype is colour‐coded in the key (right). Refer to Table S3 for specific details and location numbers.

Using the mitochondrial marker *COI* did not reveal a strong genetic structure, but some patterns regarding the distribution and diversity of haplotypes are worth noting. The 13 haplotypes plotted in Figure 5 represent 83% of the 810 *COI* sequences analysed of which 111 *COI* new sequences were generated by this work (GenBank accession numbers OK285076–OK285186). However, additional haplotypes (*n* = 99 haplotypes) are plotted in a TCS network in Figure S6. Overall nucleotide diversity (π) was 0.0256 with 75 segregating sites in our alignment. Haplotypes 2 and 3 have a global distribution and were the most prevalent haplotypes, making up 50% of all the sequences analysed (Figure 5). Haplotype 4 is widespread within the Asian and Australasian region, while similarly, H13 and H62 were only found within Asia but were less widespread. H8 and H30 are shared between parts of Asia and Africa, while H14 spans the Asian/Australasian region and Americas. Several less frequent haplotypes (Figure S6) reveal further potential structure with H92 and H95 unique to Malaysia and Indonesia, respectively.

## 4 DISCUSSION

### 4.1 Population structure

The population structure of *Ae. aegypti* across our entire study region are consistent with those of global studies (Gloria‐Soria et al., 2016), and others that share overlap with our study region (Schmidt et al., 2020). Consistent with these studies, we find that Australian populations belong to a genetic cluster distinct from Asian and Indonesian populations. This study revealed additional spatial population genetic structure of *Ae. aegypti* in Southeast Asia and Australasia compared with previous studies, particularly when populations were analysed using a hierarchical approach. Notably, within Australia, we found differentiation between multiple Queensland populations as well as between the northern Australian Waiben (Thursday Island, Torres Strait Islands) populations. When Asian‐Pacific regions have been sub‐analysed (using a hierarchical approach in STRUCTURE), Gloria‐Soria et al. (2016) found evidence that populations from Asia, Australia (Cairns and Townsville) and Pacific Ocean Islands (Hawaii and Tahiti) can be differentiated. Although samples from this study and the Gloria‐Soria et al. (2016) study were collected in different years, the years of collection are close enough that the results should be comparable (Cairns was sampled in 2013 by Gloria‐Soria et al. (2016) and 2008 in this study; Bangkok was sampled in 2013 by Gloria‐Soria et al. (2016) and 2016 in this study). Population structure uncovered in our study supports recent findings by Schmidt et al. (2020) using a large number of nuclear SNPs. In this study, we detected additional genetic breaks within Australia, Indonesia and Malaysia, as well as characterising the structure of previously unexplored populations.

Within Queensland, we found three clear genetic divisions that represent northern, south‐eastern and south‐central Queensland clusters (Figure 3), with substructure revealed with further analysis. We found a significant positive relationship between geographic and genetic distance that hints at a subtle pattern of isolation by distance, supporting the results of other studies (Endersby et al., 2009; Schmidt et al., 2020). However, the correlation (*R* ^2^) was weak, implying that other drivers such as human movements play an important role in shaping the genetic structure of *Ae. aegypti* in Queensland. In a previous study, Endersby et al. (2009) found that inland populations (Chillagoe and Charters Towers) within Northern Queensland were clearly differentiated from coastal ones, a pattern replicated in our study (see Longreach, Emerald and Charters Towers, for example). The frequency of human traffic between these Queensland populations likely dictates the amount of gene flow/genetic continuity as this increases the number of potential dispersal events. Such human‐mediated dispersal has been documented numerous times including via road, air and shipping networks (Crawford et al., 2017; Fonzi et al., 2015; Gonçalves da Silva et al., 2012; Huber et al., 2004; Powell et al., 2018; Rasheed et al., 2013; Schmidt et al., 2020). Unsurprisingly, further inland locations, such as Longreach, are more genetically isolated from easterly populations which are more connected along Queensland's major highways. As highways are a dispersal route, inland populations in Queensland may experience fewer opportunities for dispersal. Only one pairwise *F*_ST_ comparison was non‐significant, between the geographically proximate towns of Rockhampton (RO) and Mount Morgan (MT). Based on what we know about the biology of *Ae. aegypti*, it is likely that there is real population genetic structure between these sites, but that relatively small sample sizes reduced our ability to detect significant *F*_ST_ between these sites. Low genetic differentiation between these sites may be due to either gene flow or a recent shared ancestry between them.

The positive relationship between genetic distance and geographic distance is also obvious in the Waiben population from the Torres Strait Islands, which is the most disconnected Australian population from mainland Australia (both in terms of geographic distance and human traffic). Waiben would likely have higher gene flow with other island populations in the Torres Strait and Southern Papua New Guinea regions, which appears to be the case for *Ae. albopictus* (Beebe et al., 2013; Maynard et al., 2017). The two regions are highly interconnected due to the implementation of the Torres Strait Treaty which permits cross‐border movement (mostly via boat) for indigenous Torres Strait Islanders and coastal communities of Papua New Guinea and the region is also serviced by regular small aircrafts (on which other live culicids have been observed; Maynard, personal observation 2016).

The globally widespread *COI* haplotype H14 was prevalent on Waiben but was not detected in mainland Australia. This is probably reflective of the Torres Strait region differing in invasion and demographic history from mainland Australia, reinforcing earlier findings by Beebe et al. (2005). The Torres Strait Islands have a higher incidence of dengue compared to mainland Australian populations. Additionally, *Ae. aegypti* from Waiben are more competent vectors of dengue serotypes 2 and 4 compared to those from Cairns and Townsville (Knox et al., 2003), highlighting the connection between genetic structure and medically relevant traits such as vector competency. From a historical perspective, the Torres Strait Islands were part of an extensive pearling industry during the 1880s, which sought a workforce primarily from the Pacific Islands (chiefly Fiji, Vanuatu and New Caledonia), Japan, Malaya and the Philippines (Beckett, 1977). Later, during WWII, Waiben served as a military base for the United States and Australian forces. Earlier, from 1800 to 1850 many sailing ships made the voyage from Brisbane and Sydney to India and other parts of Asia via the Torres Strait, although few stopped. It would be interesting to compare Waiben to a more worldwide dataset as the Torres Strait region likely shows a different invasion history to mainland Australia.

While politically part of Indonesia, Papua is geographically and culturally connected to Papua New Guinea. Amamapare is a coastal port and cargo facility, and we found that the population from this location was genetically similar to Sulawesi and other parts of Indonesia (Bali and Sumba). This is likely due to regular migration of *Ae. aegypti* through this region via shipping movements from resulting in introgression and genetic continuity between Amamapare and other coastal Indonesian populations. In contrast, the capital of the Mimika regency, Timika (~25 km inland) was highly distinct from Amamapare (MIM) and other parts of Indonesia. The population in Timika is situated further from the coast and migration from coastal populations connected by shipping may be less frequent, but it may be more genetically similar to other unsampled inland Papuan populations. The signature from the mitochondrial *COI* dataset does not support this difference, with all Indonesian populations sharing similar haplotypes, many of which are also found across other countries, especially within Asia (Figure 5; see H2, H3, H4). However, only a subset of COI haplotypes was sampled from Papua New Guinea, Australia and the Pacific Islands (Figure 5; H2, H3, H4 and H14) with much greater haplotype diversity present in other parts of Asia and Indonesia.

In the Pacific Islands, the Honiara (Solomon Islands) population appeared more genetically distinct from the nearby population of Port Moresby than expected and is somewhat unique within our study area. This is potentially due to mixed ancestry from Australia, the USA and parts of Asia (Cambodia and Malaysia) which share the smallest pairwise *F*_ST_ measures with the population from Honiara. Human‐mediated movements during World War II would have had a significant impact on the distribution of mosquitoes in the Pacific area and could have led to admixture between geographically disparate populations (Calvez et al., 2016; Failloux et al., 2002; Powell et al., 2018). One of the most comprehensive genetic studies on *Ae. aegypti* in the Pacific (including multiple islands from New Caledonia, Fiji, Tonga and French Polynesia, but not the Solomon Islands) found moderate genetic differentiation between island populations (*F*_ST_ = 0.05–0.24) and that populations from more isolated islands were more genetically distinct than those from major towns, which showed a higher degree of mixed ancestry (Calvez et al., 2016). Nevertheless, their microsatellite data revealed population differentiation in the Pacific region, broadly corresponding with western, central and eastern genetic divisions, with further substructure within these divisions. The mtDNA markers (*COI* and *ND4*) used in their study revealed some geographic patterns of relatedness between populations, but certain haplotypes were more widespread than others. Their study highlighted that different regions likely had multiple introduction origins, both historic and contemporary. We suspect that populations from the Solomon Islands probably share some similarities to populations from other Pacific Islands (e.g. New Caledonia, Fiji, Tonga, French Polynesia) and that similar demographic processes may have occurred in the Solomon Islands. Other studies have shown that at low values of *K*, Australian populations (Townsville and Cairns) cluster with Pacific Island populations (Fiji, Vanuatu, Kiribati, New Caledonia), however, further analysis at higher values of *K* (Schmidt et al., 2019) and using other methods (Schmidt et al., 2020) supports their differentiation.

From a broader global perspective, allozyme studies have shown genetic similarities between Indonesian, Indian and Taiwanese populations (Wallis et al., 1983). Similarly, using nuclear SNPs, Schmidt et al. (2020) found that various populations of *Ae. aegypti* from Southeast Asia, Sri Lanka and Saudi Arabia belong to the same genetic cluster. This could suggest a similar introduction source, potentially revealing India as an important, yet understudied historical invasion source for Southeast Asian populations, given its importance in early trade and the early history of dengue (Gloria‐Soria et al., 2016; Smith, 1956). Using isoenzymes, Failloux et al. (2002) explored several populations including *Ae. aegypti aegypti* from French Polynesia (Southern Pacific), French Guiana (South America), SE Asia (Cambodia, Vietnam) and *Ae. aegypti formosus* from Western Africa and several Indian Ocean Islands. They found potential structure between South Pacific Island and Asian/American populations of *Ae. aegypti*, positioning African populations as more divergent. Additionally, they showed evidence of strong genetic differentiation between French Polynesian populations, which was more obvious than that between populations from Vietnam, Cambodia and French Guiana. This may have been the result of past major bottlenecks (Failloux et al., 2002) but could equally be suggestive of varying invasive origins for the isolated islands in the Pacific.

### 4.2 Invasion history in Australasia and Southeast Asia

Past notions of the worldwide spread of *Ae. aegypti* out of Africa included introduction into Asia from Africa itself. Although Africa was a major stop on voyages by British and Dutch ships travelling to Asia in the early‐1800s, genetic data‐position Asian populations as more closely related to the Americas. STRUCTURE analyses from Gloria‐Soria et al. (2016) showed that populations from the Middle Eastern region cluster with Asian and Australian populations, highlighting that the Middle Eastern region could have been an important stepping‐stone into Asia/Australasia. Overall, while there would have been ample opportunity for *Ae. aegypti* to spread from Africa to Asia, the current body of genetic evidence does not support this west‐to‐east movement.

The 16th century saw European maritime trade rise within Southeast Asia (notably for valuable spices from the Maluku [Moluccas] Islands), a region mostly under Portuguese and Spanish control, via Southern Africa (the Cape Route) and the Indian Ocean. Later in the 17th and 18th centuries, this was overrun by British and Dutch enterprises, with the Dutch monopolising the Indonesian region (under the Dutch East India Company) and possessing a widespread trade network centralised in Batavia (now Jakarta), Java. New imperialism strongly shaped trade in the 19th century, which became more globalised and began to approach today's form. Due to the frequency of human movements (especially maritime trade driven) at the time when *Ae. aegypti* established in the Indo‐Pacific, it is likely that populations from some regions in this study were established from independent introduction events.

Early records from the late‐1800s to early‐1900s, while patchy in some parts, paint a clear picture that *Ae. aegypti* colonised Asia and Australasia rapidly, and it occurred in major trading ports at a similar time (see Figure 1 and records in Table S1). James (1913) points out early concerns that the opening of the Suez Canal (in 1869) and Panama Canal (in 1914) would increase the spread of *Ae. aegypti* and associated diseases, and this were reiterated recently by Powell et al. (2018). Indeed, the opening of both canals dramatically shaped trade routes, resulting in more direct passages between Asia/Australia with the Mediterranean and the Americas. The timing of the opening of the Suez Canal in 1869 and the emergence of the first urban outbreaks of chikungunya (Carey, 1971) and dengue (Smith, 1956) shortly after support the hypothesis that this accelerated the spread of *Ae. aegypti*.

It is difficult to be sure of exact source locations of *Ae. aegypti* in Australasia and southeast Asia. This is mostly because of the possibility of reintroductions from, for example, the Americas back into Africa or the Mediterranean, from which *Ae. aegypti* could then have spread to the Asian and Australasian regions. However, accumulating genetic evidence suggests a ‘New World’ source for the species' invasion into Southeast Asia and Australasia (Gloria‐Soria et al., 2016). Recently, exome sequencing has shown that some populations in Africa appear to have been reintroduced from the Americas, providing new perspectives on the evolutionary history of populations of *Ae. aegypti* (Crawford et al., 2017). Future studies using genome‐wide SNPs will provide significant insights into the evolutionary and invasion history of *Ae. aegypti*.

### 4.3 Limitations, future work and conclusions

One of the main limitations of this study is the relatively small sample sizes for some sampling sites. This may affect estimates of allele frequency and diversity (Hale et al., 2012), as well as potentially reducing power to detect population structure. Another potential limitation of this study is the use of sampling sites as priors for DAPC analysis rather than informing priors from STRUCTURE analyses. Sampling sites were used as priors in DAPCs due to low assignment probability of many individuals in STRUCTURE analyses, suggesting admixture or shared ancestry, making it difficult to definitively assign some individuals to populations based on STRUCTURE results. Additionally, using a hierarchical approach in STRUCTURE analyses has the advantage of detecting finer scale structures that may not be apparent when analysing the entire dataset. However, it is important to be aware that once the data are separated into subsets, it will not be possible to see evidence of admixture/shared ancestry between broader groups/populations.

Microsatellites provide relatively few markers compared to what can be achieved with genome‐wide SNPs which also needs to be considered in drawing conclusions. Nonetheless, Rašić et al. (2014) found that while microsatellites can differentiate Australian, Indonesian, Vietnamese and Brazilian populations of *Ae. aegypti*, genome‐wide SNPs were far more sensitive, showing strong separation of the populations. They noted, however, that pairwise *F*_ST_ values were typically larger than that calculated from microsatellites, but that pairwise *F*_ST_ values calculated using microsatellites were often comparable across studies (Rašić et al., 2014). We expect that future studies employing genome‐wide SNPs with more comprehensive geographical sampling in the Asia‐Pacific will reveal finer scale population genetic structure and reveal more details regarding demographic histories in the region. This high level of spatial structure has been shown recently by Schmidt et al. (2019) using genome‐wide SNPs in *Ae. aegypti* from various global populations. Whether populations display seasonal differentiation in genetic structure should also be tested with such markers, however, others have found stability across the wet‐dry seasons in northern Queensland (Endersby et al., 2011) and Indonesia (Rašić et al., 2015), which might be the result of eggs surviving the dry season and hatching at the start of the wet season. As seasons are more pronounced at southerly latitudes it would be reasonable to predict that more southerly populations could undergo greater temporal genetic changes. Future genome‐wide datasets will likely uncover clearer spatial divisions within populations of *Ae. aegypti* within Australia (for instance, using landscape genomic approaches; Schmidt et al., 2019, 2020) and potentially temporal structure in regions.

Importantly, our study demonstrates the need to analyse populations of *Ae. aegypti* in the Asia‐Pacific region at a finer scale to better uncover inter‐ and intra‐continental population dynamics. This has direct implications for identifying invasion pathways for biosecurity (Endersby‐Harshman et al., 2020; Schmidt et al., 2019, 2020) and for understanding the evolutionary processes that might influence the epidemiology of *Ae. aegypti*‐borne diseases. The genetic differentiation observed between regional towns in Queensland (Australia) suggests that population removal may be possible using the incompatible insect technique. This has recently been shown to be an effective tool for suppressing *Ae. aegypti* populations in north Queensland towns (Beebe et al., 2021). This type of knowledge regarding fine‐scale population structure could be applied to other invasive insects, enabling more specific and informed decisions to be made in control management. Ultimately this would result in improved economic and public health outcomes.

## CONFLICT OF INTEREST STATEMENT

Authors have no conflict of interest.

## Supporting information


Figures S1‐S8

Table S1

Table S2

Table S3

Table S4

Table S5

Table S6

## Data Availability

All data are available through supplementary tables and new mtDNA *COI* sequences are available through GenBank (accession numbers OK285076–OK285186).
