# Supplementary material for: Population structure and invasion history of Aedes aegypti (Diptera: Culicidae) in Southeast Asia and Australasia
Source: Evol Appl. 2023 Mar 25;16(4):849–62. doi: 10.1111/eva.13541 (PMC10130559; doi:10.1111/eva.13541)
Supplement: Supplementary file 1 — Figures S1‐S8 [file EVA-16-849-s006.docx]

SUPPLEMENTARY FIGURES

**
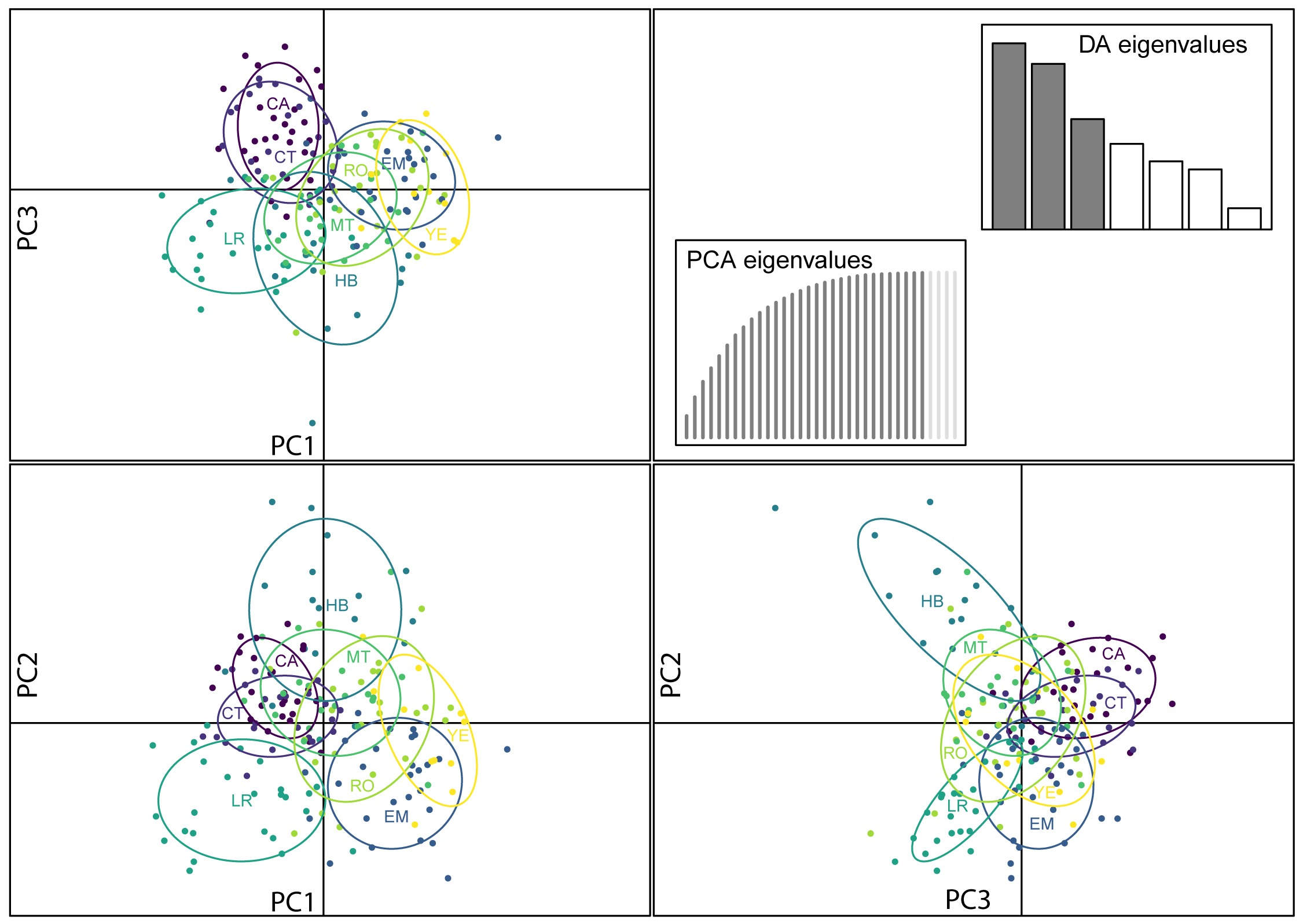
**

**Figure S1. Discriminant analysis of principal components (DAPC) for Australian populations of *Aedes aegypti* using nine microsatellite loci.** Principal components 1-3 (PC1-3) are plotted showing individual variation within populations where each point represents a separate individual and ellipses represent a populations’ 95% confidence interval. Populations are color coded and abbreviations are in Table 1.


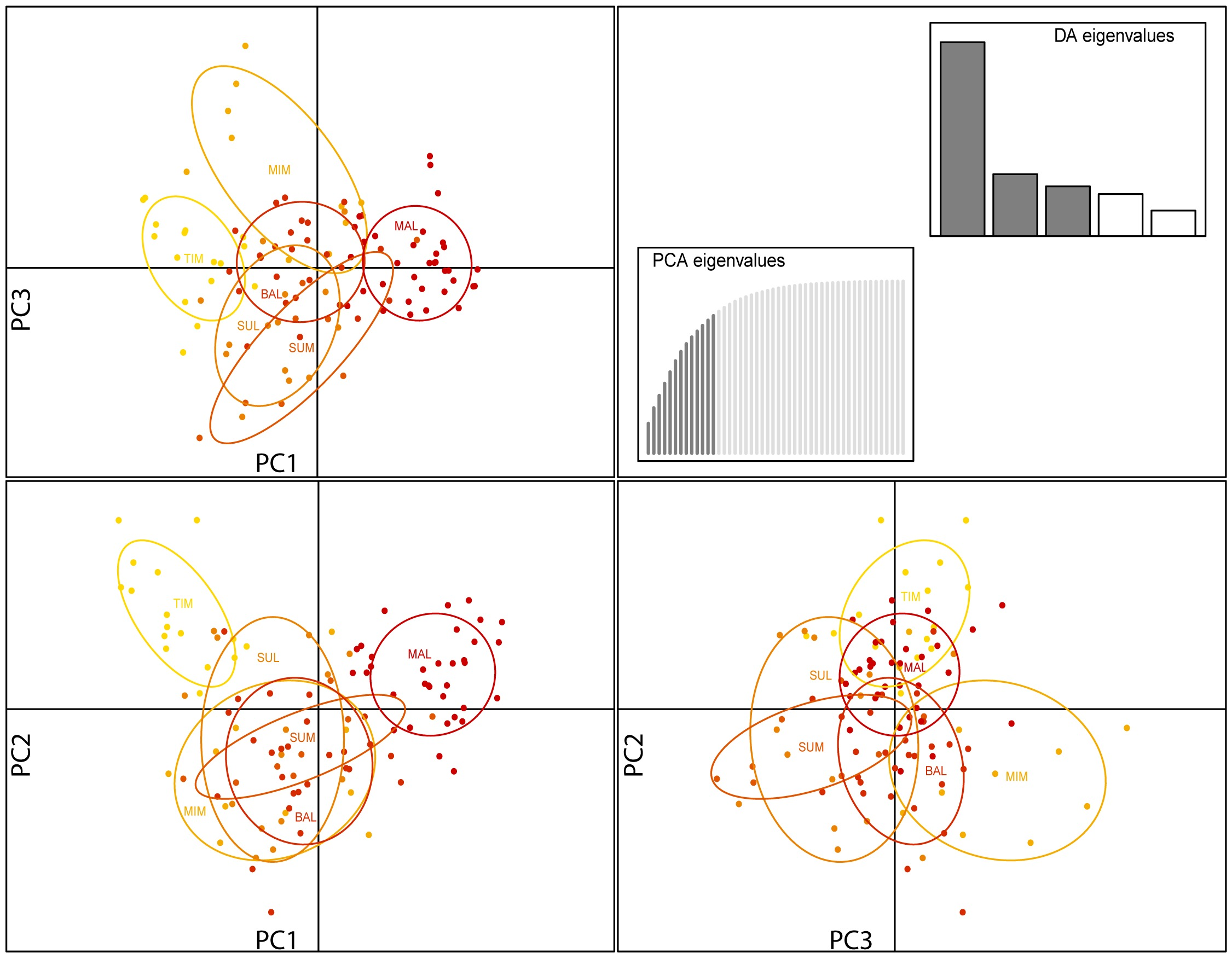


**Figure S2. Discriminant analysis of principal components (DAPC) for Indonesian and Malaysian populations of *Aedes aegypti* in our study region using nine microsatellite loci.** Principal components 1-3 (PC1-3) are plotted showing individual variation within populations where each point represents a separate individual and ellipses represent a populations’ 95% confidence interval. Populations are color coded and abbreviations are in Table 1.


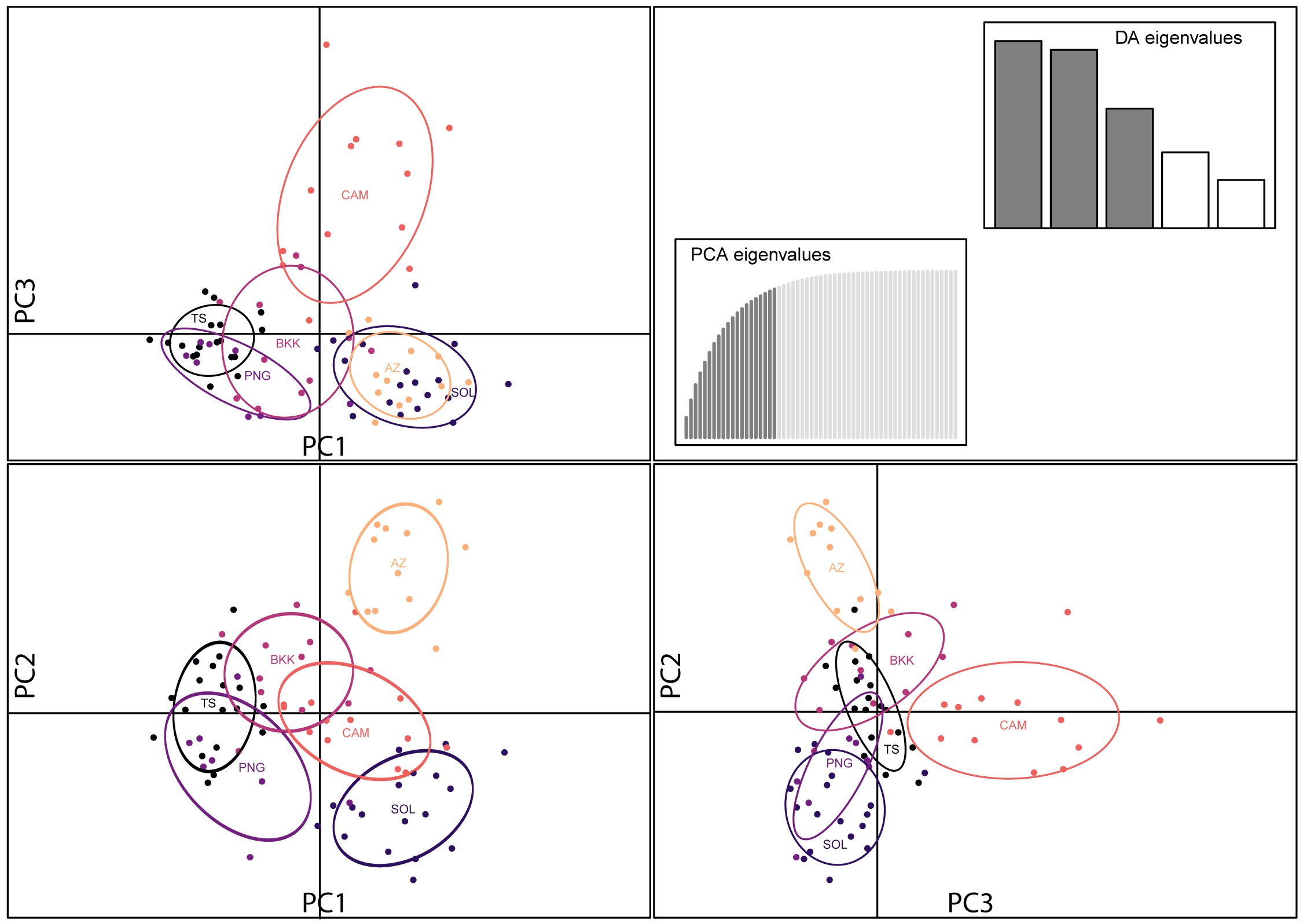


**Figure S3. Discriminant analysis of principal components (DAPC) for admixed populations of *Aedes aegypti* when K=2 in STRUCTURE analysis.** Principal components 1-3 (PC1-3) are plotted showing individual variation within populations where each point represents a separate individual and ellipses represent a populations’ 95% confidence interval. Populations are color coded and abbreviations are in Table 1.


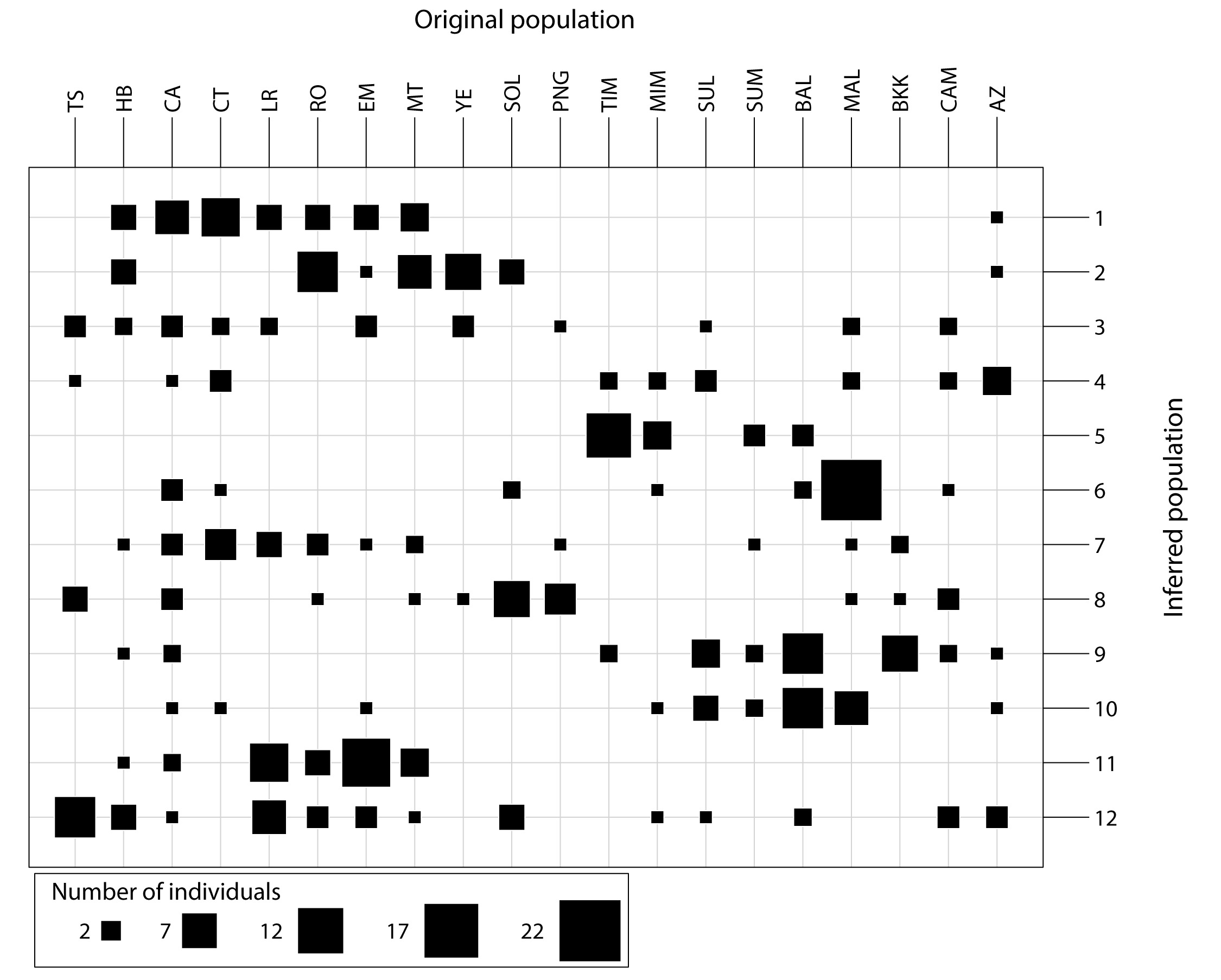


**Figure S4. Genetic clusters uncovered using the Bayesian information criterion to infer populations, given no prior population information.** Columns show the original populations (see Table 1 for population abbreviations) while rows indicate inferred populations (1-12). The scale bar shows the number of individuals assigned to each of the inferred populations.

**
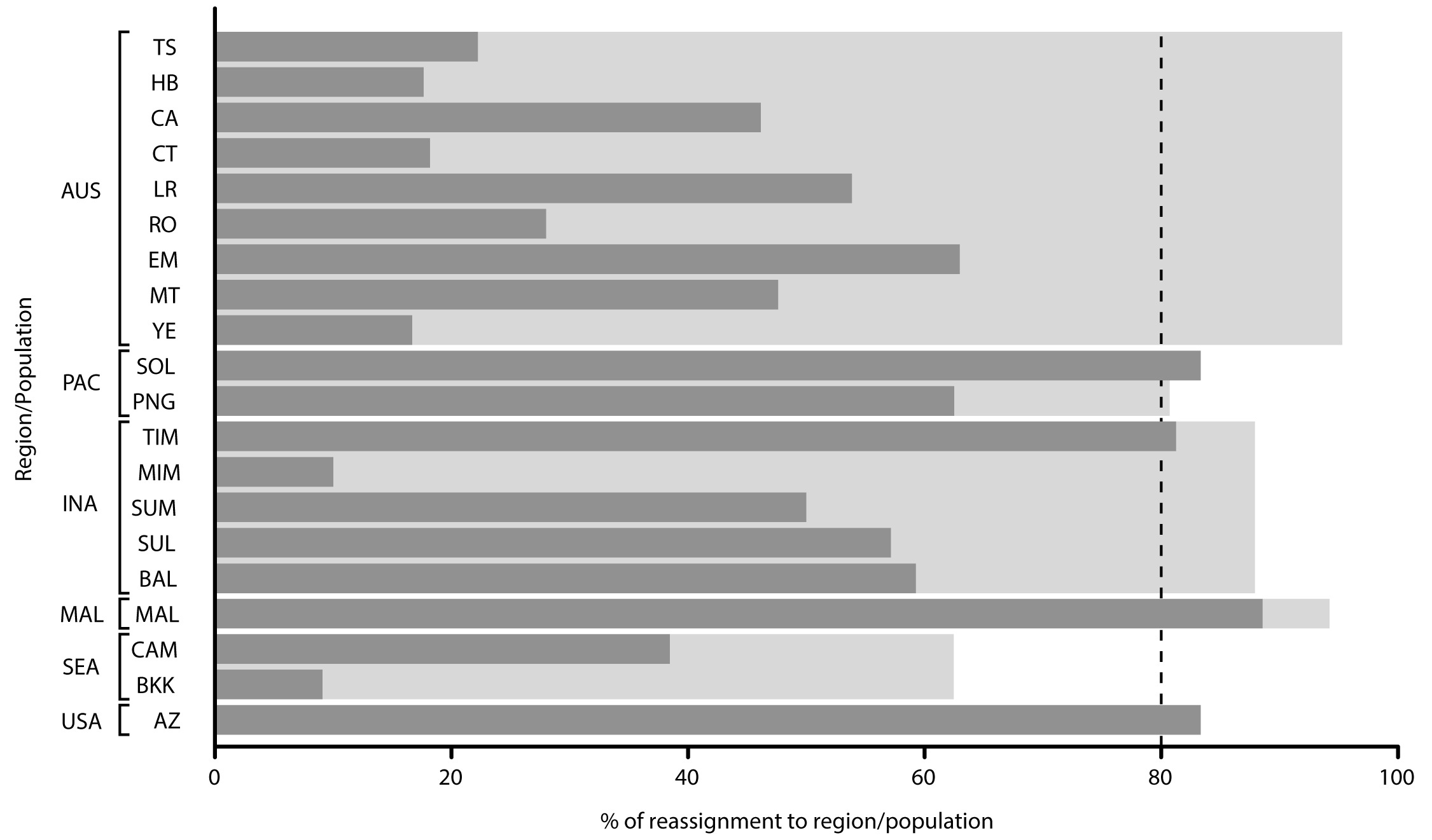
**

**Fig S5. Percentage of reassignment of individuals to original region/population based on discriminant analysis of principal components.** Dark grey bars represent a DAPC using populations while light grey bars represents a DAPC using regional definitions (see Table 1 for abbreviations). The dashed grey line represents the 80% mark which we consider indicative of strong region/population reassignment.


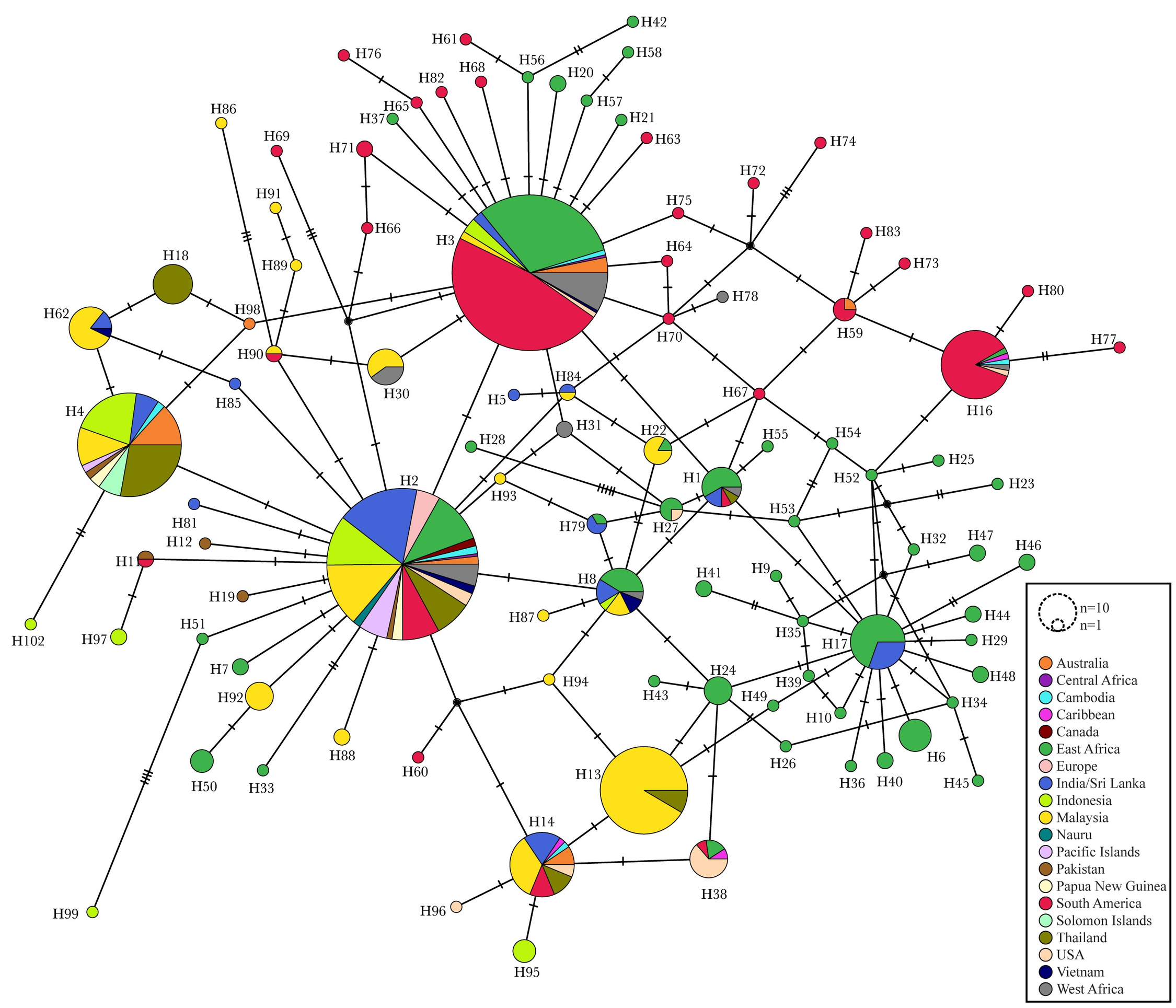


**Figure S6. Worldwide haplotype network for all *COI* haplotypes (n=99) for 810 samples of *Ae. aegypti*.** Circle size corresponds to the number of sequences (see dashed circle scale (left)) per haplotype. The proportion of individuals belonging to a given haplotype are color-coded. Refer to Table S3 for specific details.

**
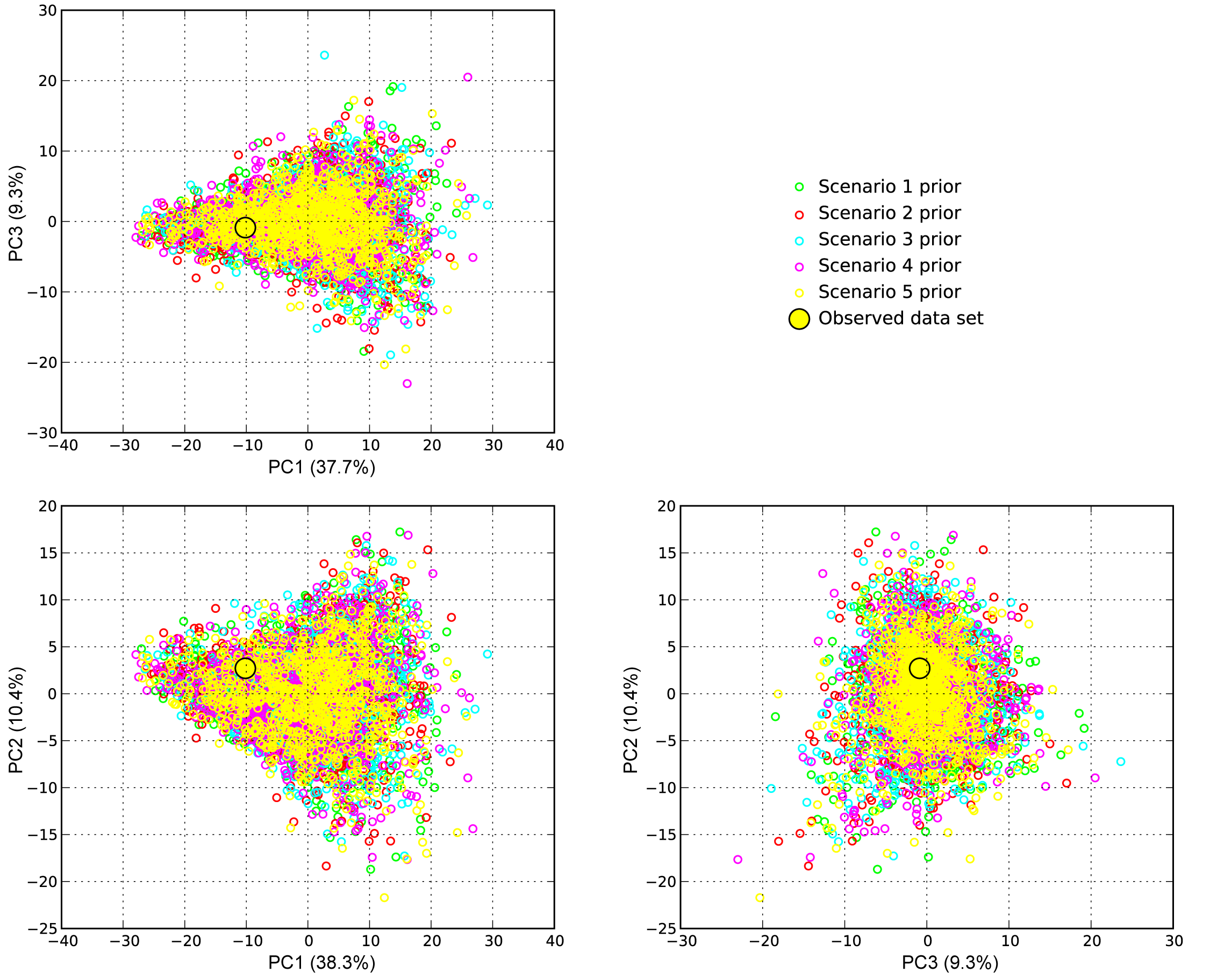
**

**Figure S7. Principal components analysis (PCA) in the space of summary statistics in DIYABC across all invasion scenarios.** The large yellow dot represents our observed dataset while colored dots show the simulated datasets for the five invasion scenarios (10,000 random prior plots displayed per scenario). The first three principal components (PC) are shown with their % variance explained by each PC shown in brackets.


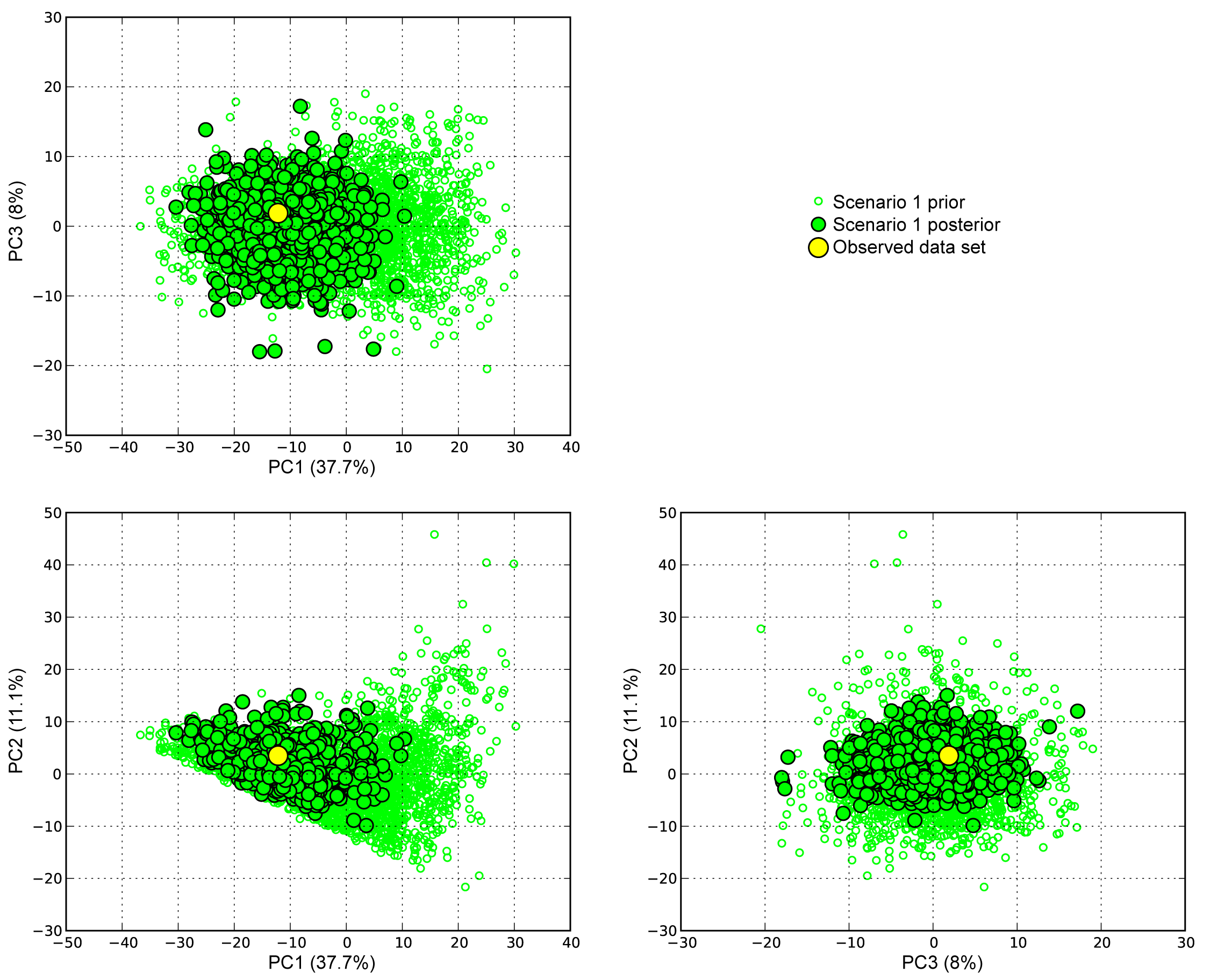


**Figure S8. Principal components analysis (PCA) in the space of summary statistics for the most likely invasion scenario (Scenario 1) in our study.** The yellow dot represents the observed *Ae*. *aegypti* dataset, solid green dots represent the simulated dataset with parameters drawn from posterior distributions (1,000 random datasets shown), while hollow green dots correspond to the datasets simulated based on prior distributions of parameters (1,000 random datasets shown). The first three principal components (PC) are plotted with the % variance explained by each PC in brackets.
